# 2,3-Bis(3-fluoro­phen­yl)tetra­zolium-5-thiol­ate

**DOI:** 10.1107/S1600536809026683

**Published:** 2009-07-15

**Authors:** Karel G. von Eschwege, Alfred Muller

**Affiliations:** a Department of Chemistry, University of the Free State, PO Box 339, Bloemfontein 9300, South Africa; b Department of Chemistry, University of Johannesburg (APK Campus), PO Box 524, Aucklandpark, Johannesburg 2006, South Africa

## Abstract

The zwitterionic title compound, C_13_H_8_F_2_N_4_S, is situated on a twofold rotation axis running along the C—S [1.691 (2) Å] single bond. The phenyl­ene ring is twisted out of the tetra­zolium plane by 42.18 (7)°. Relatively short distances [3.7572 (9) and 4.0625 (6) Å] between the centroids of the phenyl­ene and tetra­zolium rings of neighbouring mol­ecules suggest π–π inter­actions. The crystal under investigation was a non-merohedral twin, with a 33% twin component.

## Related literature

For details of the synthesis, see: Mirkhalaf *et al.* (1998 ▶); Irving *et al.* (1971 ▶). For comparison bond distances, see: Allen *et al.* (1987 ▶). For the indexing of twinned crystals by the *CELL_NOW* program, see: Bruker (2008 ▶).
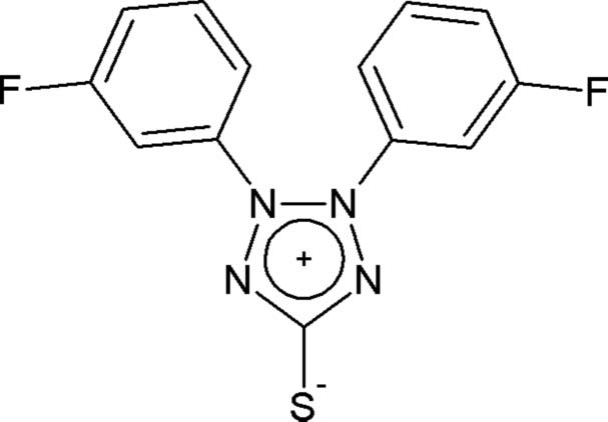


## Experimental

### 

#### Crystal data

C_13_H_8_F_2_N_4_S
*M* *_r_* = 290.29
Monoclinic, 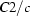

*a* = 14.500 (3) Å
*b* = 12.656 (3) Å
*c* = 6.9066 (14) Å
β = 92.93 (3)°
*V* = 1265.8 (5) Å^3^
*Z* = 4
Mo *K*α radiation
μ = 0.27 mm^−1^
*T* = 100 K
0.33 × 0.11 × 0.11 mm

#### Data collection

Bruker APEXII CCD diffractometer
Absorption correction: multi-scan (*TWINABS*; Bruker, 2008 ▶) *T*_min_ = 0.915, *T*_max_ = 0.971
1562 measured reflections
1562 independent reflections
1360 reflections with *I* > 2σ(*I*)

#### Refinement

*R*[*F* ^2^ > 2σ(*F* ^2^)] = 0.038
*wR*(*F* ^2^) = 0.101
*S* = 1.07
1562 reflections
93 parameters
H-atom parameters constrained
Δρ_max_ = 0.32 e Å^−3^
Δρ_min_ = −0.23 e Å^−3^

### 

Data collection: *APEX2* (Bruker, 2005 ▶); cell refinement: *SAINT-Plus* (Bruker, 2004 ▶); data reduction: *SAINT-Plus*; program(s) used to solve structure: *SIR97* (Altomare *et al.*, 1999 ▶); program(s) used to refine structure: *SHELXL97* (Sheldrick, 2008 ▶); molecular graphics: *DIAMOND* (Brandenburg & Putz, 2005 ▶); software used to prepare material for publication: *WinGX* (Farrugia, 1999 ▶).

## Supplementary Material

Crystal structure: contains datablocks global, I. DOI: 10.1107/S1600536809026683/ng2610sup1.cif

Structure factors: contains datablocks I. DOI: 10.1107/S1600536809026683/ng2610Isup2.hkl

Additional supplementary materials:  crystallographic information; 3D view; checkCIF report

## Figures and Tables

**Table 1:** Hydrogen-bond geometry (Å, °)

*D*—H⋯*A*	*D*—H	H⋯*A*	*D*⋯*A*	*D*—H⋯*A*
C6—H6⋯S^i^	0.95	2.79	3.6828 (19)	157

Symmetry code: (i) 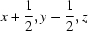.

**Fig. 1.**
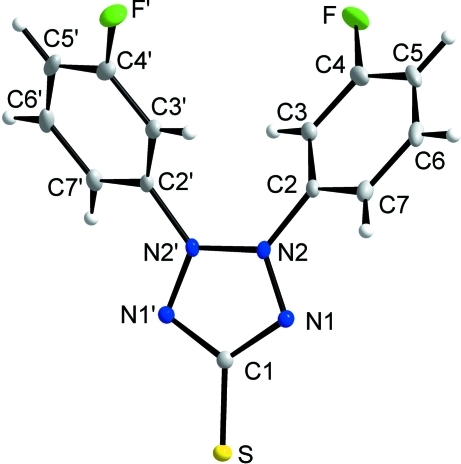
View of (I) (30% probability displacement ellipsoids). Accented lettering indicate atoms generated by symmetry (-x, y, 1/2 - z).

**Fig. 2.**
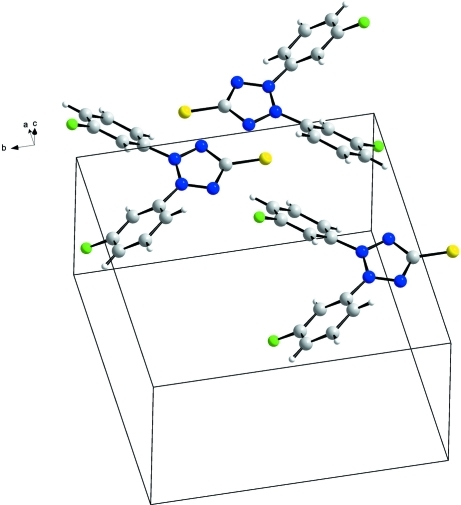
Packing diagram of (I) indicating the π-π interactions

## supplementary crystallographic information

### Comment

During the process of synthesizing a series of electronically altered dithizones for the purpose of investigating its effect on the photochromic isomerization reaction of metal dithizonates, several phenyl substituted species were fully oxidized to its dehydrodithizone derivatives. Dehydrodithizones, most probably due to their zwitter-ionic nature, crystallizes much more readily than the parent compound. The yellow *meta*-fluoro dehydrodithizone crystals, suitable for X-ray crystallography, were isolated from a mixture of polar solvents, *i.e.* acetone and water.

The title compound crystallizes in the monoclininc space group *C*2/*c* (*Z* = 4) resulting in molecules lying on special positions in the crystal lattice. All bond lengths and angles (see Table 1, Fig. 1) are within range of their expected values (Allen *et al.*, 1987). The phenyl rings adopt a non-parallel arrangement with the dehydrodithizone backbone with dihedral angles of 42.18 (7)° for ring C2—C7, mainly due to their close proximities on the tetrazole moiety. The preferred orientation is supported by the π-π stacking of the phenyl rings of neighbouring molecules (distance between planes = 3.4069 Å, centroid to centroid distance = 3.7572 (9) Å). Similar π-π stacking is also observed between neighbouring tetrazole moieties in a head-to-head fashion (distance between planes = 3.4235 Å, centroid to centroid distance = 4.0625 (6) Å) Additionally, several other close contacts/interactions are noted, among these a rather close contact for C6—H6···S between neighbouring dithizone molecules.

### Experimental

Solvents (AR) purchased from Merck and reagents from Sigma-Aldrich were used without further purification. The *meta*-fluoro derivative of dithizone, (*m*-FPhNHN)_2_CS, was prepared from ammonium sulfide and 3-fluoroaniline according to the procedure reported by Mirkhalaf *et al.*, 1998. The synthesis and crystallization of the title compound, *meta*-fluoro dehydrodithizone, was done according to a procedure reported by Irving *et al.*, 1971. Hereby a solution of (*m*-FPhNHN)_2_CS (0.3 g, 0.75 mmol) in dichloromethane (100 ml) was stirred (2 hrs) with a solution of potassium hexacyanoiron (III) (0.72 g) and potassium carbonate (0.70 g) in water (30 ml). After the organic layer was washed with water, the solvent was removed under reduced pressure. From hot acetone and water orange crystals, in 52% yield, were crystallized.

M.p 155 °C (explode). λ_max_(acetone) 434.9 nm (ε = 1650 dm^3^ mol^-1^ cm^-1^). δ_H_ (300 MHz, (CD_3_)_2_SO, 7.748 - 7.533 (8 H, m, 2x-C_6_H_4_).

### Refinement

The aromatic H atoms were placed in geometrically idealized positions (C—H = 0.95 Å) and constrained to ride on their parent atoms with *U*_iso_(H) = 1.2*U*_eq_(C). Initial CheckCIF evaluation indicated possible non-merohedral twinning, and the data was subsequantly treated using CELL_NOW to obtain orientation matrix of the two components. The raw data was then integrated as two components resulting in a HKLF5 format file, which greatly improved refinement parameters and yielded the refined composition of the twinned domains in a 33.1:66.9 ratio.

### Crystal data

**Table:**

C_13_H_8_F_2_N_4_S	*F*(000) = 592
*M**_r_* = 290.29	*D*_x_ = 1.523 Mg m^−^^3^
Monoclinic, *C*2/*c*	Mo *K*α radiation, λ = 0.71073 Å
Hall symbol: -C 2yc	Cell parameters from 1712 reflections
*a* = 14.500 (3) Å	θ = 2.8–28.2°
*b* = 12.656 (3) Å	µ = 0.27 mm^−^^1^
*c* = 6.9066 (14) Å	*T* = 100 K
β = 92.93 (3)°	Needle, red
*V* = 1265.8 (5) Å^3^	0.33 × 0.11 × 0.11 mm
*Z* = 4	

### Data collection

**Table:**

Bruker X8 APEXII 4K Kappa CCD diffractometer	1562 measured reflections
Radiation source: fine-focus sealed tube	1562 independent reflections
graphite	1360 reflections with *I* > 2σ(*I*)
Detector resolution: 8.4 pixels mm^-1^	θ_max_ = 28.4°, θ_min_ = 2.1°
φ and ω scans	*h* = −19→19
Absorption correction: multi-scan (TWINABS; Bruker, 2008)	*k* = 0→16
*T*_min_ = 0.915, *T*_max_ = 0.971	*l* = 0→9

### Refinement

**Table:**

Refinement on *F*^2^	Primary atom site location: structure-invariant direct methods
Least-squares matrix: full	Secondary atom site location: difference Fourier map
*R*[*F*^2^ > 2σ(*F*^2^)] = 0.038	Hydrogen site location: inferred from neighbouring sites
*wR*(*F*^2^) = 0.101	H-atom parameters constrained
*S* = 1.07	*w* = 1/[σ^2^(*F*_o_^2^) + (0.0484*P*)^2^ + 0.809*P*] where *P* = (*F*_o_^2^ + 2*F*_c_^2^)/3
1562 reflections	(Δ/σ)_max_ < 0.001
93 parameters	Δρ_max_ = 0.32 e Å^−^^3^
0 restraints	Δρ_min_ = −0.23 e Å^−^^3^

### Special details

**Table:**

Experimental. The intensity data was collected on a Bruker X8 Apex II 4 K Kappa CCD diffractometer using an exposure time of 180 s/frame. A total of 791 frames were collected with a frame width of 0.5° covering up to θ = 28.36° with 98.9% completeness accomplished.
Geometry. All e.s.d.'s (except the e.s.d. in the dihedral angle between two l.s. planes) are estimated using the full covariance matrix. The cell e.s.d.'s are taken into account individually in the estimation of e.s.d.'s in distances, angles and torsion angles; correlations between e.s.d.'s in cell parameters are only used when they are defined by crystal symmetry. An approximate (isotropic) treatment of cell e.s.d.'s is used for estimating e.s.d.'s involving l.s. planes.
Refinement. Refinement of *F*^2^ against ALL reflections. The weighted *R*-factor *wR* and goodness of fit *S* are based on *F*^2^, conventional *R*-factors *R* are based on *F*, with *F* set to zero for negative *F*^2^. The threshold expression of *F*^2^ > σ(*F*^2^) is used only for calculating *R*-factors(gt) *etc*. and is not relevant to the choice of reflections for refinement. *R*-factors based on *F*^2^ are statistically about twice as large as those based on *F*, and *R*- factors based on ALL data will be even larger.

### Fractional atomic coordinates and isotropic or equivalent isotropic displacement parameters (Å^2^)

**Table:**

	*x*	*y*	*z*	*U*_iso_*/*U*_eq_	
S	0	0.63767 (5)	0.25	0.01916 (16)	
N1	0.07681 (9)	0.44217 (11)	0.27936 (18)	0.0170 (3)	
N2	0.04565 (8)	0.34445 (11)	0.26789 (18)	0.0156 (3)	
F	0.10346 (9)	0.01182 (11)	0.57483 (18)	0.0443 (4)	
C1	0	0.50409 (18)	0.25	0.0158 (4)	
C2	0.10317 (10)	0.25414 (13)	0.3084 (2)	0.0179 (3)	
C3	0.07274 (12)	0.17451 (14)	0.4266 (2)	0.0212 (4)	
H3	0.0134	0.1766	0.4786	0.025*	
C4	0.13285 (14)	0.09224 (16)	0.4647 (3)	0.0284 (4)	
C5	0.22040 (14)	0.08883 (16)	0.3976 (3)	0.0321 (5)	
H5	0.2606	0.0316	0.4304	0.039*	
C6	0.24883 (12)	0.17000 (16)	0.2820 (3)	0.0310 (4)	
H6	0.3092	0.1687	0.2346	0.037*	
C7	0.18989 (11)	0.25393 (15)	0.2339 (2)	0.0243 (4)	
H7	0.2087	0.3094	0.1522	0.029*	

### Atomic displacement parameters (Å^2^)

**Table:**

	*U*^11^	*U*^22^	*U*^33^	*U*^12^	*U*^13^	*U*^23^
S	0.0192 (3)	0.0169 (3)	0.0216 (3)	0	0.00337 (19)	0
N1	0.0156 (6)	0.0179 (7)	0.0174 (6)	−0.0005 (5)	0.0011 (4)	−0.0009 (5)
N2	0.0111 (6)	0.0197 (7)	0.0158 (6)	−0.0011 (5)	0.0000 (4)	−0.0007 (5)
F	0.0556 (8)	0.0311 (8)	0.0447 (7)	0.0079 (6)	−0.0118 (6)	0.0152 (5)
C1	0.0159 (10)	0.0193 (11)	0.0123 (9)	0	0.0025 (7)	0
C2	0.0142 (7)	0.0195 (8)	0.0193 (7)	0.0017 (6)	−0.0046 (5)	−0.0042 (6)
C3	0.0209 (8)	0.0219 (9)	0.0199 (8)	0.0016 (7)	−0.0062 (6)	−0.0019 (6)
C4	0.0329 (11)	0.0239 (10)	0.0268 (9)	0.0044 (8)	−0.0121 (7)	−0.0001 (7)
C5	0.0283 (10)	0.0281 (11)	0.0379 (10)	0.0139 (8)	−0.0175 (7)	−0.0120 (8)
C6	0.0165 (8)	0.0369 (11)	0.0388 (10)	0.0066 (7)	−0.0067 (7)	−0.0182 (8)
C7	0.0165 (8)	0.0281 (10)	0.0281 (9)	0.0001 (6)	−0.0006 (6)	−0.0085 (7)

### Geometric parameters (Å, °)

**Table:**

S—C1	1.691 (2)	C3—C4	1.375 (3)
N1—N2	1.3177 (18)	C3—H3	0.95
N1—C1	1.3685 (18)	C4—C5	1.374 (3)
N2—N2^i^	1.334 (2)	C5—C6	1.377 (3)
N2—C2	1.434 (2)	C5—H5	0.95
F—C4	1.352 (2)	C6—C7	1.393 (3)
C1—N1^i^	1.3685 (18)	C6—H6	0.95
C2—C7	1.383 (2)	C7—H7	0.95
C2—C3	1.383 (2)		
			
N2—N1—C1	104.75 (13)	F—C4—C5	119.28 (18)
N1—N2—N2^i^	110.19 (8)	F—C4—C3	117.58 (18)
N1—N2—C2	122.82 (12)	C5—C4—C3	123.14 (19)
N2^i^—N2—C2	126.72 (8)	C4—C5—C6	118.76 (18)
N1^i^—C1—N1	110.1 (2)	C4—C5—H5	120.6
N1^i^—C1—S	124.94 (10)	C6—C5—H5	120.6
N1—C1—S	124.94 (10)	C5—C6—C7	120.60 (17)
C7—C2—C3	122.81 (16)	C5—C6—H6	119.7
C7—C2—N2	117.37 (15)	C7—C6—H6	119.7
C3—C2—N2	119.74 (14)	C2—C7—C6	118.11 (18)
C4—C3—C2	116.54 (17)	C2—C7—H7	120.9
C4—C3—H3	121.7	C6—C7—H7	120.9
C2—C3—H3	121.7		
			
C1—N1—N2—N2^i^	0.36 (17)	N2—C2—C3—C4	−177.74 (14)
C1—N1—N2—C2	−173.99 (11)	C2—C3—C4—F	−177.94 (14)
N2—N1—C1—N1^i^	−0.14 (7)	C2—C3—C4—C5	2.5 (3)
N2—N1—C1—S	179.86 (7)	F—C4—C5—C6	178.54 (16)
N1—N2—C2—C7	−43.6 (2)	C3—C4—C5—C6	−1.9 (3)
N2^i^—N2—C2—C7	143.01 (18)	C4—C5—C6—C7	0.0 (3)
N1—N2—C2—C3	133.08 (15)	C3—C2—C7—C6	−0.5 (2)
N2^i^—N2—C2—C3	−40.3 (2)	N2—C2—C7—C6	176.04 (14)
C7—C2—C3—C4	−1.2 (2)	C5—C6—C7—C2	1.2 (2)

Symmetry codes: (i) −*x*, *y*, −*z*+1/2.

### Hydrogen-bond geometry (Å, °)

**Table:**

*D*—H···*A*	*D*—H	H···*A*	*D*···*A*	*D*—H···*A*
C6—H6···S^ii^	0.95	2.79	3.6828 (19)	157

Symmetry codes: (ii) *x*+1/2, *y*−1/2, *z*.
